# Photography and Medicine

**Published:** 1936

**Authors:** A. E. Iles

**Affiliations:** Surgeon to the Bristol Eye Hospital; Ophthalmic Surgeon, Bristol General Hospital


### The Bristol Medico-Chirurgical Journal

*"Scire est nescire, nisi id me*
*Scire alius sciret."*

SPRING, 1936.

### PHOTOGRAPHY AND MEDICINE.

The Presidential Address, delivered on 9th October, 1935, at the opening of the Sixty-third Session of the Bristol Medico-Chirurgical Society.

BY

MR. A. E. ILES, O.B.E., M.B., F.R.C.S.,
*Surgeon to the Bristol Eye Hospital;*
*Ophthalmic Surgeon, Bristol General Hospital.*

THIS year nearly coincides with the centenary of the invention of Photography, and is the actual centenary of the first negative which is still in existence; and bearing in mind the great strides which have been made in photography in connection with Medicine, I consider that it would not be out of place to trace the history of this process from its inception, dealing particularly in the later stages with the co-operation and help that photography has given and should continue to give to Medicine.

### THE DEVELOPMENT OF PHOTOGRAPHY.

Prior to 1519 Leonardo da Vinci had made the first camera obscura, which had a pin-hole aperture, and in 1568 Danielo Barbaro discovered that the brightness and definition of the images were improved by means of a lens and diaphragm. But it was not until Scheele in 1777 observed that moist silver chloride was darkened by light, and that the blue and violet rays of light had the most effect that the photo-sensitiveness of silver salts was investigated. Senebier a few years later showed that violet rays had as much effect on moist silver chloride in fifteen seconds as red rays had in twenty minutes.

The photo-sensitiveness of silver salts had been experimented upon by Thomas Wedgwood (fourth son of Josiah Wedgwood, the famous potter), who suffered from ill-health due to an obscure malady and turned to science. In 1802 he read a paper with Sir Humphry Davy before the Royal Institution entitled "An account of a method of copying paintings upon glass, and of making profiles, by the agency of light upon nitrate of silver." No further progress was made at the time. This was partly due to Wedgwood's early death three years later at the age of thirty-four, and partly because they were unable to fix the images so obtained, being unaware of the discovery by Chaussier in 1799 that hyposulphite of soda stabilized these images. This salt was not brought into regular use until Sir John Herschel advised its employment in 1839. In 1810 Seebeck of Jena discovered that by throwing a true spectrum by means of a properly constructed prism on to moist silver chloride for fifteen or twenty minutes in the region exposed to the violet rays the silver chloride turned reddish-brown, while the part exposed to the blue rays gave a clear blue fading to a lighter blue in the region exposed to the green, the portion acted upon by the yellow was unaltered, whilst the part acted upon by the red or infra-red became rose or lilac. This paper was the first mention of the different colour effects of light upon silver chloride and was the basis of most experiments in colour photography.

The first real progress was made by Henry Fox Talbot experimenting in 1834 at Lacock Abbey in Wiltshire, and by the Niepces, father and son, working with Daguerre in France. Talbot and Daguerre worked independently upon the sensitiveness of silver compounds to light, and their methods, chemicals and reactions were quite different. Although Daguerre's name is associated with the process, known as "Daguerreotype," Niepce in 1826 had been working on making images for some time, and in 1829 a deed of partnership was drawn up between Niepce and Daguerre in which Niepce disclosed his process to Daguerre: the latter had only made some improvements in the camera obscura. Niepce died in 1833, and there is no doubt that Niepce's work was the foundation of Daguerre's process, and that until Daguerre obtained from Niepce the details of his process he (Daguerre) had made no advance at all in obtaining fixed images.

Daguerre first used an emulsion of asphaltum in oil of lavender on a metal plate, but he found that with this method the necessary time of exposure of a landscape in ordinary daylight was from seven to eight hours. He then used a silver-surfaced copper plate and exposed it to iodine vapour: this formed silver iodide, which was his sensitive material. This plate was immediately placed in the camera and exposed; it was then taken out and developed in a closed chamber with a window in it so that the action of vapourized mercury on the plate could be watched and development stopped when the image had been sufficiently brought out: the images so obtained were extremely brittle and had to be handled with great care. Fizean in 1840 removed this disability by toning with gold chloride and hyposulphite, and this is the first mention of toning in connection with photography. The time of exposure was appalling—twenty to thirty minutes in blazing sunlight: the length of time necessary and the suffering of sitters in consequence were the butt of the French caricaturists of that period. Goddard in 1840 cut short the exposure by his discovery that the addition of bromine to the iodine brought the time down from minutes to seconds.

Fox Talbot's process was entirely different. Starting in 1834, he from the first relied upon the double process of negative and positive, and although he did not patent the method until 1841, there is in existence a negative, from which prints can still be made, of a window in Lacock Abbey taken in 1835. He called his method the calotype process, and in the hands of experts like D. O. Hill very good portrait studies were obtained. He used paper which was first coated with potassium iodide and then sensitized by a solution of gallic acid and silver nitrate. After exposure the paper was developed in a further bath of gallic acid and silver. He first of all used sodium iodide or chloride as a fixing agent, but quickly gave it up for hyposulphite of soda. After washing and drying the paper was waxed in order to render it more transparent, and from this negative any number of contact prints could be made. Owing to the fact that Fox Talbot used paper for his negatives there was always a certain amount of grain in the prints: this, although making a certain type of print more attractive, was considered a handicap for scientific purposes and compared poorly with the sharp, clear-cut image obtained by Daguerre's process. Eventually, in order to get rid of the grain, glass was used for the negatives, being coated in the first place by an albumen emulsion.

The Rev. J. B. Reade also had experimented in photography before Daguerre's method was disclosed in 1839. His was in some way an improvement on Fox Talbot's process, as he used gallic acid in the coating, thus increasing the sensitivity of the emulsion; he was, however, of a retiring disposition and did not publish his method; he was probably the first to use hyposulphite of soda for fixing, as in April, 1839, he had shown pictures taken by his process and fixed in this way.

We see, therefore, that although Daguerre's process has practically gone into disuse the method and principle of Fox Talbot's invention has been the basis of our further developments. Both with Fox Talbot's and Daguerre's processes the time of exposure was far too long. The next step was the wet collodion process of Archer in 1850. He obtained an emulsion on a glass plate by means of an iodized solution of pyroxyline dissolved in alcohol and ether: this formed a transparent coating when the solvents had evaporated. This plate was soaked immediately before use in a solution of silver nitrate, with formation of silver iodide: the plate was exposed when wet and then immediately developed with a solution of pyrogallic acid or sulphate of iron made acid by acetic acid. This wet process remained in favour for more than thirty years because it gave grainless negatives combined with greatly increased speed of exposure, but it necessitated a portable dark room and permanently blackened fingers because of the necessary constant dipping in the silver bath. Some of these negatives were developed up to a white image, the glass plate being then backed with black varnish. This gave results comparable with those of the Daguerreotype, but at a fraction of the cost and also of the time of exposure. Many of the photographs which are still in existence in small leather cases and thought to be Daguerreotypes are in reality photographs by this method.

The next improvement as regards the negative was the introduction by Dr. R. L. Maddox of Southampton of the gelatine dry plate in 1871. This gave a still greater impetus to photography, as the time of exposure was for the first time brought to a fraction of a second. Sensitive dry plates were not, however, put on the market until 1877, these continuing the only usual medium for negatives until the introduction of transparent roll film by George Eastman in 1889.

With regard to positives. Paper positives remained stabilized for a long period until Blanquart-Evrard observed that by coating the paper with albumen the image was prevented from sinking in. This paper had to be sensitized by floating on a bath of silver nitrate, dried and used in a day or so. Ost discovered in 1870 that by adding citrate to the sensitizing bath the paper could be kept for months both before and after printing. This albumenized paper remained the medium for positives until 1891, when Messrs. Ilford introduced a gelatine emulsion, which was a great advance as it kept good much longer, and in consequence rapidly took the place of the too often ill-smelling albumenized paper which had been generally imported.

Next as regards early cameras. Those used by Fox Talbot were quite small, taking prints only two inches square, this being due to the fact that no large photographic lenses existed, and so he had to use lenses from his microscopes and telescopes which had only a focal length of one to two inches. This led to Andrew Ross making longer focus lenses for him, and in 1841 Petzval worked out the formula which is the basis for the manufacture of portrait lenses of to-day.

One can gauge the improvement by the length of exposure necessary :

| | |
|---|---|
| Daguerreotype .. .. .. | 30 minutes. |
| Calotype .. .. .. .. | 2–3 minutes. |
| Wet collodion process .. .. | 10–15 seeonds. |
| Rapid gelatine emulsion .. | 1/15th second. |
| Ultra-rapid kinematography .. | 1/100,000th of a second. |

Photographs taken by the latter method were demonstrated this year (1935) before the Royal Photographic Society.

### THE USE OF PHOTOGRAPHY IN MEDICINE.

At first the main use of photography in medicine was that of recording cases and abnormalities, of course in black and white. The discovery of Röntgen rays in 1895 gave a great impetus to the use of photography in medicine, since by the aid of X-ray "photographs" the site and nature of a fracture of a bone could be accurately determined; and when the fracture had been set a further skiagram showed the surgeon whether the two ends of the fractured bone were in apposition or no.

As improvements in technique and apparatus followed so did these "photographs" become more and more useful to the clinician: the use of substances opaque to these rays, such as barium salts, lipiodol, opacol, etc., enlarged the field still more, since movements of the stomach and bowel could be accurately watched, obstructions, dilatations and growths displayed to be observer, cavities in the lung, the size and position of the heart, and the state of bony cavities such as the accessory nasal sinuses could be demonstrated, the condition of the roots of the teeth determined and the presence made out of stones in gall-bladder, bladder and kidney. Ventriculography has also been of use in the study of cerebral tumours and disease, and lately thorotrast injections into the carotid artery and subsequent skiagraphy have shown aneurysms of the circle of Willis and other vascular intracranial lesions and by the displacement of the arteries the position of a brain tumour.

Dr. Russell Reynolds in 1934 demonstrated a method of X-ray cinematography by which means the movements of the heart, joints and bowel can be accurately studied and any deviation from the normal made manifest. X-rays have also been of service in the study of the vessels of the heart: injections of an opaque medium and then subsequent skiagraphy have demonstrated the ramifications of the cardiac vessels.

Stereoscopic X-rays are useful in the examination of the relation of the fœtal head to the pelvis, also in the accurate localization of the position of foreign bodies. Sweet's method of localization of foreign bodies in the orbit is definitely valuable.

The introduction of cinematography, and particularly the 16mm. film, greatly extended the sphere of usefulness of photography. Already an extensive film library of different medical subjects has been formed by Messrs. Kodak, in which the steps of an operation or the paths of infection in certain cases can be projected upon the screen. In America, where the method of teaching is somewhat different from ours, lectures are made more valuable, as the student can follow the steps of an operation as it is being described. I myself have been endeavouring to get the different types of nystagmus on a strip of film for demonstration purposes, but as so frequently happens the cases which I required have been conspicuous by their absence. Since slow motion cinematography has been introduced, the study of certain gaits, such as those of tabes, the intention tremor of disseminated sclerosis, the methods of reduction of a dislocation can be readily demonstrated, and by its use in obstetrics the mechanism of such unusual cases as face and brow presentations can be visually presented to the student, who otherwise would not have such an opportunity unless he was so especially fortunate as to be a midwifery student when such a case turned up. Cinematography is also of service in illustrating the methods and steps of orthopædic treatment. Dr. Forrester Brown has already given the Society such a demonstration.

The cardiologist has also been aided in his study by the introduction of the electro-cardiograph, which has enabled him to observe the cycle of the heart's movements.

I have so far dealt with the black and white method of photography, but with the advance of research colour photography has made its appearance. Some time ago colour transparencies by Lumière's method were available, but these necessitated the use of fairly dense filters and long exposures. Improvements in this branch have steadily been made, and there are now several fairly satisfactory methods of colour cinematography and transparencies available which only require a little increase of exposure over that necessary for the black and white, though the method of giving one direct positives in colour has not yet made its appearance for prints.

Photography by means of the penetrating power of infra-red rays has already proved to be of definite service to medicine. In lupus the infra-red photograph does not show the scabs which one sees in the panchromatic negative; consequently the dermatologist can estimate the amount of active disease present without having to remove the scabs. Infra-red photographs also show congested and tortuous veins below the surface in the breasts of pregnancy, also in the legs of patients with varicose ulcers. Dekking of Nymwegen has shown how the use of this method is of direct service to ophthalmology, in that it enables one to obtain a good photograph of the iris through a turgid cornea, this being useful in cases of interstitial keratitis, where it is of advantage to know how effectively atropine is acting upon the iris.

Photo-micrography as a branch of photography has considerably advanced in usefulness to medicine, as the quality and definition of lantern slides and positives has greatly increased, partly due to the advent of the panchromatic process and also partly due to improvements in the colour transparencies. A photo-micrograph of a section which a class is studying can by means of a lantern slide be thrown upon the screen, so that the teacher can demonstrate step by step the salient points of the section, while the students are able to follow him whilst observing sections from the same block through their microscopes. Minute examination of serial sections can be carried out by positives laid side by side, and two years ago a demonstration of serial sections by cinematography was given, though I myself should prefer the slower method of examination, as the cine film would tend to pass too quickly to be of any practical use.

Lantern slides in colour also help to demonstrate pathological lesions. Infra-red photography is also of service for lantern slides and photo-micrographs, particularly in cases of asbestosis of the lung, as the infra-red ray passes through the chitinous envelope and displays the fibre more clearly.

The use of ultra-violet rays has been of most service in medico-legal work, where stains on clothing, etc., only faintly visible to the naked eye and by ordinary photography, are brought out markedly by the use of ultra-violet photography. This method is especially useful in detecting seminal and faint blood stains.

The Nordensen camera has been in use for photography of the fundus oculi for some time now, but the objection to its use was that the prints were in black and white, they were small and consequently had to be enlarged. Experiments are now being carried out in order to ascertain whether colour transparencies taken by this method would be of service: I am showing one of the early trials by this method.

Fincham in 1935 showed photographs of the lens in accommodation and rest, taken by means of slit lamp illumination in an aniridic eye. These show very well the position of the ciliary processes and the shape of the lens in rest and accommodation. Since then he has taken photographs of the crystalline lens of a child of eleven, and by means of section of the zonula the different curves of the lens are distinctly shown; he has also by means of photography been able to demonstrate the position of Purkinje's figures in rest and accommodation.

I have endeavoured to show in this address how great a field is open to photography in the study and recognition of disease in widely-different branches of medicine.

Medico-legal investigation is aided by the use of ultra-violet photography; dermatology by the use of infra-red photographs and also by colour transparencies, as the colours now obtained are practically true and the time of exposure necessary is not too long. Surgery benefits by the use of ordinary photographs such as those of dislocations, by the study of efficient X-ray photographs, the study of the movements of joints and intestines by X-ray cinematography. Cinema studies in normal and slow motion of the methods of reduction of dislocations should be of value, also by ventriculograms and X-rays after thorotrast injections in cerebral disease. Cardiology is aided by the use of X-rays, X-ray cinematography, and the electro-cardiogram. Pathology and research is helped by the study of micro-photographs of serial sections, also by the use of lantern slides both in panchromatic, colour and infra-red. Ophthalmology by infra-red, fundus photography, and the study of the lens and ciliary processes by means of slit lamp photography. Medicine is greatly assisted by X-ray photography, and could be further aided by the use of cinema films illustrating by normal and slow motion gaits, tremors and nystagmus, and the study of the pathology of the case by means of photo-micrographs. I well remember the great collection of advanced tabetics and other cases of disease of the central nervous system that the late Dr. Michell Clarke had attending his out-patients: such advanced cases are rarely seen nowadays, whereas a good cinematograph study of these gaits would have ensured a permanent record being kept of them, and so they would not have been lost to us.

In conclusion, I hope that I have brought forward sufficient evidence to show that every university and large teaching hospital should be provided with an efficient photographic department, equipped with the necessary cameras and apparatus, and the provision of a properly qualified technician in attendance (photo-micrography would almost in itself ensure that his time would be fully occupied). I am sure that if this were provided our study of disease would be materially benefited and case records would become more valuable, especially if at the same time a proper photographic library were provided with adequate indexing, so that any particular case could be looked up and examined with facility. These records, I am positive, would be of considerable value both at the present time and to posterity, more so as visual memory is in the main much more retentive and reliable than auditory memory.

